# Interprofessional student-led clinics: the volunteer patient experience

**DOI:** 10.1186/s12909-022-03760-6

**Published:** 2022-10-11

**Authors:** Annette Burgess, Chris Roberts

**Affiliations:** The University of Sydney, Faculty of Medicine and Health, Sydney Medical School, Education Office, 2006 Sydney, Australia

**Keywords:** Interprofessional, Patient safety, Student-led clinic, Peer learning

## Abstract

**Background:**

Learning from patients and gaining an understanding of their lived experience plays an important role in improving health professions education. However, opportunities for students to engage in interprofessional learning activities involving patients as partners remain limited. In 2018, we developed an interprofessional student-led clinic where people living with Parkinson’s Disease voluntarily participated as ‘patient-partners’. The aim of this pilot study was to explore patients’ experience and motivation for participation.

**Methods:**

In 2018 the clinic was implemented five times. Four patient volunteers and six to eight students from a mix of disciplines attended each clinic. Qualitative data were collected via semi-structured focus groups with patients. Data were analysed using thematic analysis.

**Results:**

Eleven patients participated in the focus groups. Patients found the interprofessional nature of the clinic beneficial to their health goals. Their interactions with students from different disciplines helped to build their healthcare knowledge and confidence to ask additional questions of health professionals. Patients felt they offered unique perspectives to students of their own lived experiences. They found sharing their stories with students and each other built a sense of community.

**Conclusion:**

Patients felt they enriched the learning environment, helping students to build their knowledge and skills by providing authentic patient perspectives. The interprofessional aspect enhanced the patient experience in a number of ways. Patients found the multiple perspectives of healthcare helped them to build their own knowledge, and reflect on their changing needs. Warranting further investigation, our findings indicate that participation in the clinics may have positively influenced patients’ health seeking behaviours.

## Background

The long-held tradition of teaching with the involvement of real patients lies at the heart of medical education. Learning from patients and gaining an understanding of their lived experience plays an important role in improving the education and training of health professionals. Many errors in healthcare arise from not listening to the patient voice and poor interprofessional teamwork [1]. Collaborative, interprofessional practice forms the cornerstone of safe patient care, relying on health professionals interacting and communicating effectively with all healthcare team members, including the patient. Generally, students find encounters with patients in the clinical setting to be authentic and meaningful, leading to improvements in ease of communication, building of rapport, and appropriate expressions of empathy [2–5].

In recent years, an increasing emphasis has been placed on the inclusion of patients and their carers/caregivers in healthcare dialogue, providing greater transparency, and growing recognition of the role of interprofessional care [6, 7]. Yet opportunities for students to engage in interprofessional learning activities involving patients as partners within the clinical setting remain limited [1, 6, 8]. Recent studies by Oates & colleagues described changes in attitudes of junior health professionals following participation in a patient safety course for emerging leaders that was interprofessional and involved learning from patients [1, 7]. Participants noted the ‘presence of patients to provide authentic stories’, initiated ‘motivation to foster change in the workplace and a greater awareness of patient-centred care’ [1]. Other known benefits for students include development of communication and clinical skills, and the opportunity to learn from patients who are experts of their own illness [9].

Many studies have demonstrated positive patient experiences when involved in student teaching, noting a desire to contribute to student education [10–12]. However, less is known about the patient experience when voluntarily participating in student-led interprofessional clinics unrelated to patient care. In this pilot study, we sought to explore patients’ experience and motivation to participate in a student-led, interprofessional clinic, where people living with Parkinson’s Disease voluntarily participated.

## Methods

### Study context

In 2018 a student-led, interprofessional clinic was developed and piloted at a large University teaching hospital. It was delivered five times across the year. Each clinic was four hours in duration. In total, 32 senior students participation throughout 2018. All students were either in their final or penultimate year of study, enrolled in a graduate-entry or undergraduate health professional program. Six to eight senior students from a mix of disciplines (pharmacy, medicine, physiotherapy, occupational therapy, or speech pathology) participated in each clinic.

Four people living with Parkinson’s Disease voluntarily attended each clinic. They were recruited from the local branch of a Parkinson’s patient support group. All patients were taking anti-Parkinson’s medication, and had access to a neurologist and general practitioner. Two had a mild disability with onset of Parkinson’s in the last 18 months, eight were moderate, and one was severe and largely wheel chair bound. They all spoke English. Following the student-led clinic and lunch, some of the patients attended a therapy session at the hospital. One educator and one clinician supervised each clinic.

#### Learning activity

Students were provided with a 30min orientation by the lead academic for the day, and provided with the Parkinson’s Disease Clinic General Assessment Form to guide the patient assessment. This form had been previously developed by an interprofessional practitioner team for a local Parkinson’s Clinic Service run once per month by an aged care consultant. In uniprofessional teams of two, students rotated through four ‘stations’, spending 30min with each volunteer patient, taking a history and performing an examination. The first pair of students would see that the patient established their three health goals that they wanted to achieve in the clinic. Once all students had spent time with each volunteer patient, they then attended a 30min interprofessional team meeting. Together, students were required to produce an integrated patient management plan to present to the clinical supervisor.

### Study design

#### Data collection and analysis

Qualitative data were collected from patients at three of the five clinics. At the end each clinic, all patients were invited to participate in a focus group. Semi-structured focus group questions were used, with questions based on the patient experience and perceptions of participating in the clinic. Data were transcribed verbatim. Thematic analysis was used to build an understanding the patients’ experience of the interprofessional student-led clinic. Each author (AB and CR) first made familiarization notes regarding the emergence of themes in the data. We then met to discuss the coding, and identified broad patterns within the transcript. Following negotiation between authors, a coding framework was developed [13].Both authors reviewed, defined and named the themes. We then chose data excerpts that best represented each theme for a more in-depth analysis. To resolve any disagreements that arose in interpretation, we discussed issues until consensus was achieved.

#### Reflexivity

Reflexivity refers to the identification of the researchers’ beliefs and assumptions and understanding of relevant topics during the process of conducting qualitative research [14]. Discussions between the authors encouraged reflexivity. The different perspectives and insights on the data as an educationalist and a general practitioner helped to support the interpretation.

## Results

Eleven ‘patients’ (five female and six male) participated in the focus groups. In exploring the perspectives of the patients, five primary themes were identified that characterised their experience.

Theme 1: Interprofessional aspect.

Theme 2: Reflecting on their health condition, and changes over time.

Theme 3: Increased knowledge and confidence to engage in healthcare and seek further advice.

Theme 4: Desire to help the health professionals of the future/teach students.

Theme 5: Social aspect.

### ***Theme 1: Interprofessional aspect***

Patients felt that communicating with students from across various disciplines helped them to build knowledge of their own healthcare needs.

*Everyone has got something to add to what you want to know. They all look at it differently.*

*You get to look at perspectives of the whole thing - some seem to do more than others, but it depends what they’re trying to find out from you.*

Patients felt they benefit from participating in the clinic because it reminds them to ask about things they may have forgotten, or their doctor has not spoken about to them.

*- - - these different clinicians [from various disciplines], they ask pertinent questions of us, things that perhaps, you might forget about, and then you sort of think to yourself, ‘Oh yes. That’s sort of important, and that’s happened to me.’*

Patients were open to students from the various disciplines discussing different health aspects.

*I did listen intently… and they all asked you a lot of questions, maybe in a different roundabout way with each group [discipline], but they still asked you a lot of questions and listened. We talked about the combination of medications. I asked questions, they gave me a little bit of advice about a couple of things and I found it very useful. I found it very useful, and the physiotherapists were very good too, challenging, but good.*

### ***Theme 2: Reflecting on their health condition, and changes over time***

Patients found it beneficial when talking with students, to reflect on their experience of changes in their health condition over time. They felt this helped them to be mindful of continuing to try and improve their quality of life.

*Even just talking to them. You can relate back, how you were one or two years ago to what you’re like now…it’s not until you look at what you were then to what you are now you realise how much you may have changed. Otherwise you… wouldn’t even think about it. You think, oh yeah, I’m just a bit worse, and just carry on…. I used to do this two years ago. Now I can’t. When did it change? and what can I do to… get your muscles back.*

*I have a dreadful memory short term, so that is the hardest part for me. So that’s good in here. It teaches just little things that – that I’ve forgotten about.*

Patients felt that participating in the clinic made them more proactive to gain different perspectives.

*I thought, oh, hang on… I haven’t thought about for so long, but yes, it has happened….so that’s how they help us. I seem to think more about Parkinson’s than we would have done generally… because we’re getting different perspectives.*

They indicated that they learnt and wanted to learn more from students.

*They pushed me harder …and physically more than the others. But I noticed I’ve got a bit slower than when I started..… it will come back. I would like to find a little bit more about the exercises.*

*Yeah, they recommended the speech pathologist to look for the swallowing and for my voice. They suggested I join a choir…*

### ***Theme 3: Increased knowledge and confidence to engage in healthcare and seek further advice***

The patients shared experiences of increased confidence to engage in healthcare as a result of participating in the clinic. They felt they may benefit from the clinic because students may have new ideas.

*Ah, because it’s Parkinson’s, and it relates to us, and anything that can help us is worthwhile. Even if it’s only one thing. Because we’ve all got a thing that’s particular to you that you have trouble with, and if you can get that sorted out …it’s one that we haven’t done before.*

*They’re the new ones coming on. They might have new ideas. Things that haven’t even been thought of.*

They felt it helped them identify their own limitations and how to maintain health.

*I think it’s worthwhile for my own sake for me to know what I can do and what I can’t do and I know the limitations I’ve got, but - because I’ve never had them tested.*

*We are benefitting by this trial. We are…It’s not just the students….at the same time - - - We come out of it learning something ourselves. So please, keep it going as long as you can. We come out of it learning something ourselves.*

### ***Theme 4: Desire to help the health professionals of the future/teach students***

Patients assist students in their learning by offering their own unique experiences of Parkinson’s Disease and how it has impacted their lives.

*I look like I don’t have any symptoms at all, and that’s something that they need to understand. Well sitting here now and looking at the four of us, you wouldn’t know any of us have got Parkinson’s.*

Patients felt they were contributing to student learning regarding Parkinson’s, and that they could offer unique assistance with this from their lived experiences.

*People say to me ‘oh but you look so good’. Yeah, that’s ‘cause I’m managing my symptoms … from a student point of view, they have to understand that it’s also not just the Parkinson’s itself, it’s the associated illnesses that happen when you develop Parkinson’s…. the Parkinson’s won’t kill you, it will be something associated that’s linked through Parkinson’s. *

Patients feel by participating in the clinic they can raise awareness - they want people to understand the range of presentations.

*I believe no two people seem to have the same symptoms in Parkinson’s. Some are similar, but not exactly the same. The more we learn about it and what’s happening, people are aware of it, the better as far as I’m concerned, because people see us shaking and thinking, what’s wrong with him, you know. And yet their memory in their head is good, but their body has gone.*

They were happy to be able to help students in their training as health professionals, and felt they had unique and diverse perspectives to offer that couldn’t be gained elsewhere.

*It’s experience for them. Face-to-face contact with people, understanding. The best way to point out what Parkinson’s is all about, is to get beside patients…. someone that’s got it. Someone’s that’s experienced the problem. And various problems. And there’s a diverse lot of symptoms here, we’re all different.*

*I think the more face-to-face time they can have, then they get more patient, better understanding, and it jogs them to think okay, well this person’s got this symptom, but hasn’t got this one. It gets them more of a knowledge of a baseline to work with, because … we’re all diverse, no one of us is the same. And we’ve all got different medication too and different strengths, different levels.*

Patients feel they are able to offer different stages of Parkinson’s disease and show students that signs are not always obvious

*we don’t walk around with Parkinson’s disease - - - - tattooed on our forehead…..I think they would be more aware and because they’re seeing such a – a different diversity of Parkinson’s.*

### ***Theme 5: social aspect***

Patients felt the clinic was an additional means to meet with others who live with Parkinson’s disease.

*I think it’s a good idea, because then you might run into people …. that have got Parkinson’s, and you can relate to it. I think the more knowledge is good for us and meeting, but that’s my social side of it too.*

## Discussion

In this pilot study, we sought to investigate the volunteer patients’ perspective and motivation to participate in an interprofessional student-led clinic. Analysis demonstrated that patients valued the experience. They felt the interprofessional nature had associated benefits, such as increasing their own knowledge of various healthcare aspects. Patients were motivated by a desire to shape student learning and a sense of community.

Patients felt that the interprofessional nature of the clinic helped them to build their own knowledge of Parkinson’s disease and understanding of how it impacts their lives. Literature suggests that reasons for negative patient experiences during teaching encounters include student disinterest, and exclusion from communication between the student and the supervisor [15–17]. However, patients involved in this clinic were able to shape the quality of their own experience, and what they learnt from different disciplines. They felt benefits included learning from the various disciplines, and reflecting on what they may be able to try in order to improve their own lives. Incorporating the priorities and preferences of patients in their care is an important aspect of ‘person-focused care’ within the interprofessional context, with the best information available from patients [18, 19].

Following participation in the student-led clinic, patients felt empowered in personal health-related behaviours. The incorporation of the patient’s priorities in their care was emphasised at the student orientation session and the interprofessional team meeting, which may have contributed to this outcome. It has been suggested that adequate briefing of students is particularly important prior to patient interaction, contributing to patient engagement and satisfaction [9]. Further, a recent systematic review by Mangin and colleagues to identify studies of existing instruments available to record patient preferences and priorities for care, highlighted an urgent need to develop ways to make patient priorities explicitly visible in clinical records, and in medical decision making [19]. They also highlighted the importance of visibility of patient priorities across disciplines and multiple treatment providers [19].

Patients indicated that interactions with students from various disciplines during the clinics helped to build their knowledge and confidence to ask additional questions of healthcare professionals. Another study highlighted the therapeutic nature of discussing their illness with students [9].

Patients were motivated by an altruistic desire to grow the capacity of the healthcare workforce by offering their unique input into student education and training. They wanted to increase student awareness of the variety and stages of presentations of Parkinson’s Disease. Patients were able to contribute to both students and other patients in constructing knowledge and understanding of Parkinson’s Disease, such as recognising signs, symptoms and different stages. By participating in the clinic they were able to offer unique perspectives that can only be learnt by listening to the voice of individual patients and their experiences. Our findings are in line with current literature showing that involving patients in teaching, where they share the experience of their illness, may increase their sense of empowerment and satisfaction [15, 16, 18]. A study conducted by Thistlethwaite and Cockayne (2004) to investigate the attitudes of patients being interviewed by first year medical students showed that most patients felt they had benefited from the process, finding it useful and interesting [9]. Our study also demonstrated that patients found the clinic offered a means for them to meet with each other, share their stories, and increase their sense of community. Similar studies have reported that participating in health professional student education increases willingness for future involvement with students [20].

## Limitations

The strength of our study was being one of the first to provide qualitative insights into the patient experience of an IPE experience of patients living with a chronic neurological disease. We acknowledge that this is a pilot study, and that our findings may not be generalisable to other contexts. Although no unwanted negative experiences were identified by patients, this is an important aspect to consider.

## Conclusion

Patients felt they enriched the learning environment, helping students to build their knowledge and skills by providing authentic patient perspectives. The interprofessional aspect enhanced the patient experience in a number of ways. Patients found the multiple perspectives of healthcare helped them to build their own knowledge, and reflect on their changing needs. These aspects might be considered by educators in designing programs that are supportive of volunteer patients. Increasing our understanding of the patient experience and their motivation to participate as patient partners may assist future patient recruitment. Warranting further investigation, our findings indicate that participation in the clinics may have positively influenced patients’ health seeking behaviours.

## Data Availability

Datasets supporting the conclusions of this article are included within the article. The datasets generated and analysed during the current study are not publicly available due to confidentiality agreements approved by the Human Research Ethics Committee, but are available from the corresponding author on reasonable request.
